# Functional Magnetic Resonance Imaging in the Final Stage of Creutzfeldt-Jakob Disease

**DOI:** 10.3390/diagnostics10050309

**Published:** 2020-05-15

**Authors:** Stefan M. Golaszewski, Bettina Wutzl, Axel F. Unterrainer, Cristina Florea, Kerstin Schwenker, Vanessa N. Frey, Martin Kronbichler, Frank Rattay, Raffaele Nardone, Larissa Hauer, Johann Sellner, Eugen Trinka

**Affiliations:** 1 Department of Neurology, Christian Doppler Medical Center, Paracelsus Medical University, 5020 Salzburg, Austria; b.wutzl@salk.at (B.W.); c.florea@salk.at (C.F.); k.schwenker@salk.at (K.S.); v.frey@salk.at (V.N.F.); raffale.nardone@gmail.com (R.N.); j.sellner@salk.at (J.S.); e.trinka@salk.at (E.T.); 2 Karl Landsteiner Institute for Neurorehabilitation and Space Neurology, 5020 Salzburg, Austria; 3 Neuroscience Institute, Christian Doppler Medical Center, Paracelsus Medical University, 5020 Salzburg, Austria; m.kronbichler@salk.at; 4 Institute for Analysis and Scientific Computing, Technical University of Vienna, 1040 Vienna, Austria; frank.rattay@tuwien.ac.at; 5 Institute of Neuroanesthesiology, Christian Doppler Medical Center, Paracelsus Medical University, 5020 Salzburg, Austria; a.f.unterrainer@salk.at; 6 Centre for Cognitive Neuroscience and Department of Psychology, University of Salzburg, 5020 Salzburg, Austria; 7 Department of Neurology, Franz-Tappeiner-Hospital, 39012 Merano, Italy; 8 Department of Psychiatry, Psychotherapy and Psychosomatic Medicine, Christian Doppler Medical Center, Paracelsus Medical University, 5020 Salzburg, Austria; l.sellner@salk.at; 9 Department of Neurology, Landesklinikum Mistelbach-Gänserndorf, 2130 Mistelbach, Austria; 10 Department of Neurology, Klinikum rechts der Isar, Technische Universität München, 81675 München, Germany

**Keywords:** Creutzfeldt-Jakob disease, functional magnetic resonance imaging, coma, apallic syndrome, alinetic mutism, sensorimotor cortex, vibration paradigm

## Abstract

Sporadic Creutzfeldt-Jakob disease (sCJD) is a rare fatal degenerative disease of the central nervous system. The clinical course is characterized by rapid progression of neurological and neuromuscular symptoms. The late stage with loss of consciousness is not well characterized. We report a 62-year-old male patient with sCJD with the clinical picture of a vegetative state/apallic syndrome, in whom we studied cortical responses using a vibration paradigm. The functional magnetic resonance imaging (fMRI) investigation demonstrated a clear response within the sensorimotor cortex, the cerebellum, the parietal cortex, the insular, and frontal inferior region. The finding of persistent cortical activity on fMRI in a patient with CJD in a state of unconsciousness has implications for the clinical management and for ethical considerations.

## 1. Introduction

Sporadic Creutzfeldt-Jakob disease (sCJD) is a lethal neurodegenerative disease with mortality rates of 70% within the first year of diagnosis [1]. The clinical manifestations are heterogenous, but patients eventually develop to akinetic mutism/apallic syndrome/vegetative state in the advanced disease course [2]. 

The evaluation of patients with disorders of consciousness in combination with progressive neuropsychiatric and neuromuscular signs and symptoms, however, is challenging. To overcome the limitations of the clinical examination, the guidelines from the European Academy of Neurology (EAN) recommends a multimodal evaluation of these patients with electroencephalography (EEG)-based techniques and functional neuroimaging [3]. 

Functional magnetic resonance imaging (fMRI) is an emerging tool to evaluate covert consciousness and cortical networks in patients with vegetative state [4,5,6]. Since clinical evaluation of patients with CJD frequently implicate that cortical functions are absent in advanced CJD disease, we challenged this hypothesis with a somatosensory fMRI paradigm in a comatose patient with sCJD. The abundance of self-awareness in CJD patients in the final stage is likely to have ethical impact on clinical decisions and management.

## 2. Case Report 

### 2.1. Clinical Course and Ancillary Findings 

A 62-year-old male with unremarkable medical, neurological, and family history was admitted with progressive cognitive deterioration, behavioral disturbances, and social regression which were noticed five months earlier. Neurological examination showed an intention tremor at both upper extremities, parkinsonian symptoms with rigidity and additional spastic symptoms of the extremities, the trunk, and bulbar muscles. There was severe ataxia in gait and targeted movements. Systemic rhythmic myoclonus of trunk and face muscles were noted. He had expressive and perceptive aphasia with symptoms of additional disturbances of higher brain functions (agraphia, acalculia). His state of consciousness was reduced, although awake. The nest kin signed the approval for the MRI examination. The ethical committee provided a waiver for anonymized retrospective reporting (25 September 2019, Ethics Committee of Salzburg, Austria 415-EALL/5/36-2019).

**Figure 1:**
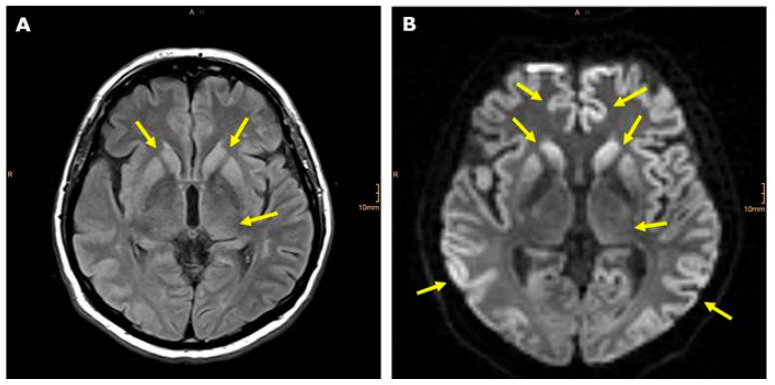
(**A**) Axial Fluid-attenuated inversion recovery (FLAIR)-weighted magnetic resonance imaging (MRI) shows hyper-intense basal ganglia and left thalamus. (**B**) Diffusion-weighted imaging (DWI) revealed a restriction of diffusion within the bitemporo-parietal and bifrontal region of the cerebral cortex, and again within basal ganglia and left thalamus (yellow arrows).

Fluid-attenuated inversion recovery (FLAIR)-weighted MRI revealed hyperintensive signals (yellow arrows) within the left thalamus (dorsomedial and lateral posterior nucleus) and within the right and left caudate nucleus and putamen (Figure 1A). Hyper intensities (yellow arrows), as a sign of restricted diffusion, of frontal, temporal, and parietal lobes, as well as left thalamus and right and left caudate nucleus and putamen were detected in diffusion-weighted MRI (Figure 1B). MRI spectroscopy showed a decreased N-aspartyl-aspartate (NAA)/ choline (CHO) ratio. Repeated EEGs revealed general slowing, a left temporal focus, bilaterally synchronized sharp waves periodically almost throughout the entire recording with a frequency of 1.5 to 2 Hz, that are sometimes configured triphasically, and an encephalopathic pattern. Total-tau and 14-3-3 protein were elevated in the cerebrospinal fluid (CSF). Cortical malperfusion was detected by single photon emission computed tomography (SPECT) in the parietal lobes, but not in the temporal lobes. The 99mTc-HMPAO (hexamethylpropylene amine oxime) and Ioflupane (123l)-SPECT did not show any signs for Parkinson’s or Alzheimer’s disease, respectively. Then, we suspected CJD. 

In the following two months, a rapid deterioration of the clinical conditions was observed and ended in an apallic state seven months from symptom onset. Respiratory failure occurred and the patient died four weeks after onset of coma. Brain autopsy showed findings consistent with prion disease. Briefly, mild spongiform changes were noted in the temporal cortex, basal ganglia, cerebellum, thalamus, and parietal cortex, whereas the changes were moderate in the frontal cortex, hippocampal formation, and occipital cortex. The presence of pathological prion protein was demonstrated immunohistochemically as diffuse synaptic deposits in the frontal cortex, temporal cortex, basal ganglia, cerebellum, occipital cortex, and parietal cortex. Mild to moderate neuronal loss was observed in these regions. Reactive astrogliosis and microgliosis were moderate to severe. The presence of amyloid plaques, neurofibrillary tangles, or α-synuclein-positive inclusions could not be demonstrated by immunohistochemistry. Further details are shown in Table 1.

### 2.2. Coma Scale and fMRI

With the intention to assess cortical functions, an fMRI was performed four weeks before death, and one week after onset of coma. The coma recovery scale (CRS) on the day of neuroimaging as well as the previous days repeatedly revealed an apallic state. 

**Figure 2:**
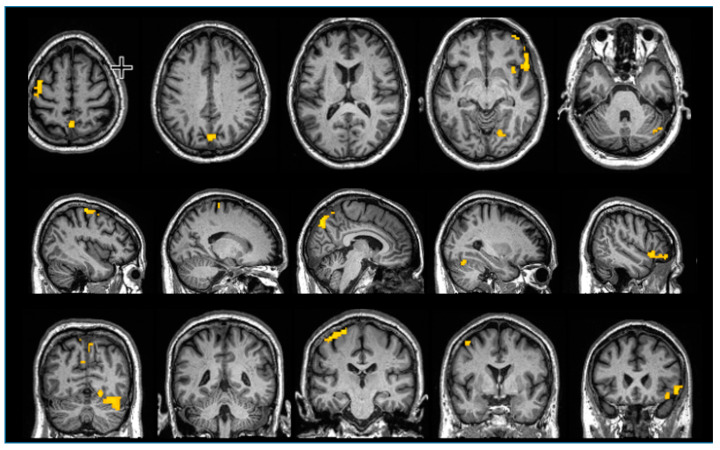
Functional magnetic resonance imaging (fMRI) of the patient with pneumatic finger vibration on the right thumb and index finger. Blood oxygenation level dependent (BOLD) response is seen contralaterally within the primary sensorimotor cortex SM1, within the right cerebellar hemisphere, bilaterally within the posterior parietal somatosensory cortex, and within the insular and inferior frontal cortex of the right hemisphere.

As can be seen from Figure 2, vibrotactile stimulation of the right hand resulted in statistically significant enhanced blood oxygenation level dependent (BOLD) signal in a cluster located in the contralateral left hemisphere sensorimotor cortex, within the right cerebellar hemisphere, bilaterally within the posterior parietal somatosensory cortex, and within the insular and inferior frontal cortex of the right hemisphere.

### 2.3. Anesthesiological Preparations and Procedures

Due to unresponsiveness, agitation, and myoclonus, the patient was planned for fMRI scan under general anesthesia. Because of expected airway difficulties after extubation, fully monitored overnight surveillance in an isolated room was scheduled. Setup for anesthesia was prepared according to In’s article [7]. Non-ferromagnetic standard monitoring (Precess, Invivivo Corp., Orlando, FL, USA) was connected. Non-ferromagnetic plastic laryngoscope, ventilation bag, facemask, O_2_ flow meter, endotracheal tube, and stethoscope were of single use type. Single use filters were inserted between the valve and the endotracheal tube. The MRI suitable ventilator Parapac (Pneupac Ltd, Luton, UK) was protected against contamination by a plastic overlay. The ferromagnetic infusion pumps were deposited outside the MRI room, the line was extended to 4.5 meters.

Anesthesia was induced with propofol 200 mg and maintained with propofol 5.5 mg/kg^−1^, because sevoflurane preferentially modulates higher-order connections [8]. Analgesia was provided initially by fentanyl 0.15 mg and two repetitive doses of fentanyl 0.1 mg. Intubation was facilitated with rocuronium 80 mg. Systolic blood pressure was kept over 100 mm Hg by infusing phenylephrine 10 mg solved in 500 mL of Ringer’s solution. The patient was ventilated with an FiO_2_ of 0.45 to an end-tidal CO_2_ of 37mm Hg. SpO_2_ was 99% throughout the whole anesthesia. Extubation and recovery was uneventfully. The whole equipment for anesthesia was incinerated, except the infusion pump and the ventilator.

### 2.4. MRI Parameters

The analysis was performed on a 32-channel head coil on a Siemens Magnetom Trio Tim scanner (Erlangen, Germany). During each of the 3 functional runs, 104 (excluding the 6 dummy scans) whole brain images were obtained with a T2*-weighted single-shot echo-planar sequence (TR 2200 ms, TE 30 ms, FA 70°, FOV 210 × 210 mm; matrix 64 × 64). Additionally, a high resolution anatomical image was acquired with a 3D MPRAGE T1w sequence (TR 2300 ms; TE 2.91 ms, TI 900 ms, FA 9°; 160 slices; slice thickness 1.20 mm; in-plane resolution 1.0 mm × 1.0 mm; GRAPPA = 2). During each run, which started and ended with a rest (no vibrotactile stimulation) epoch, 7 rest epochs alternated with 6 vibrotactile stimulation epochs, both epoch types lasted for 16 secs [9,10,11], during which, vibrotactile stimulation was applied to the index and the middle finger of the patient’s right hand.

SPM12 Software (http://www.fil.ion.ucl.ac.uk/spm/) was used for data preprocessing with additional in-house matlab scripts and tools from Analysis of Functional NeuroImages (AFNI) and Functional MRI of the Brain Software Library (FSL). Data Preprocessing consisted of brain extraction using FSL’s brain extraction tool (BET), realignment and distortion correction using a fieldmap, despiking using AFNI’s 3ddespike function, slice-timing correction and spatial smoothing using 6mm FWHM Gausssian Kernel. The T1w-structural image was normalized into MNI-template space via the CAT12-toolbox by segmenting and denoising the image and warping MNI-space via the DARTEL normalization algorithm. These normalization parameters were used to warp the functional images into MNI Space after co-registering it to the unnormalized T1w image. 

The patient’s fMRI data were analyzed using a General Linear Model which included each vibration epoch convolved with a synthetic Hemodynamic Response Function (HRF) in a block design and the six realignment parameters as covariates to control for residual noise due to head movements. Furthermore, a high-pass filter (128 s cutoff) was used to remove low frequency drifts and temporal autocorrelations were modelled with an AR(1) process. A one-sample t-contrast was used to identify voxels with higher BOLD for vibration compared to rest epochs and thresholded at *p* < 0.001, uncorrected, at the voxel level and controlling for multiple comparisons via cluster-level correction of *p* < 0.05, family-wise error (FEW)-corrected.

## 3. Discussion

The novel finding reported in the present paper consists of a task-dependent activation of the contralateral left primary sensorimotor cortex, the right cerebellar hemisphere, the posterior parietal somatosensory cortex, and the ipsilateral insular and inferior frontal cortex. The widespread network of cortical activation was detected in a CJD patient studied in the late stages of the disease, presenting clinically with the full picture of a comatose state, which, in the further course of the disease, developed into an apallic syndrome. 

This finding is usually not detected in other conditions characterized by severe impaired level of consciousness, such as hypoxic–ischemic encephalopathy [12]. This is highly surprising, since the cortex is involved early in the course of the disease [13,14]. Therefore, despite the typical rapid progression of the neurological signs, the cerebral cortex maintained widespread activity within the sensorimotor brain network, until the later stages of the disease. The neuronal pathways studied by fMRI are projected to a large extent to the parietal cortex that is, however, less frequently affected by spongiform changes. Interestingly, the neuropathological examination detected pathological prion protein in that region. Our findings implicate that, in the final stage of CJD, there are still functional afferent somatosensory neuronal pathways that are addressed by the applied fMRI paradigm with functional projections to the postcentral gyrus, and the posterior parietal region where BOLD response could be elicited in the fMRI investigation.

General anesthetics at a deep sedative dose preferentially reduce brain activation in higher-order information-processing regions, but leave the reactivity of primary sensory cortices to stimuli unchanged [15,16,17]. Accordingly, we hypothesized that, during light anesthesia with propofol, there would be reduced BOLD activity in memory-related and higher-order information-processing brain regions, while BOLD response within primary somatosensory information-processing brain areas should remain unchanged. However, we cannot rule out any influence of the applied anesthesia on the BOLD response due to the applied vibration with absolute certainty. 

It should be noted that we found this BOLD-response exclusively within the sensorimotor brain network, because other functions could not be examined in this study. This activity disclosed by fMRI could suggest the persistence of cortical plasticity, and therefore, the theoretical possibility of a functional recovery. Obviously, the clinical and coma recovery scale revised (CRS-R)-based diagnosis of an apallic syndrome did not prove true according to our fMRI investigation. 

## 4. Conclusions 

In conclusions, the fMRI examination of a comatose patient with CJD who was clinically rated as in an apallic state showed evidence for preserved cortical activity. Thus, fMRI could be used as a tool to support a more precise characterization of the patient´s state in advanced CJD. Moreover, the assessment of cortical activity could serve as a biomarker in upcoming clinical trials.

## Figures and Tables

**Table 1:** Distribution of the pathological alterations in the brain.

Brain Region	Spongiform Changes	Prion IHC	Neuronal Loss	Astrogliosis	Microgliosis
Frontal cortex	++	+	++	+++	+++
Hippocampus	-	-	++	++	++
Temporal cortex	+	+	++	++	++
Parietal cortex	+	+	+	++	++
Occipital cortex	++	+	++	+++	+++
Basal ganglia	+	+	+	++	++
Thalamus	+	-	+	++	++
Cerebellum	+	+	+	+	+
Substantia nigra	-	-	-	+	+
Pons	-	-	+	+	+

+: rare, ++: moderate, +++: extensive.
